# Analysis of Tks4 Knockout Mice Suggests a Role for Tks4 in Adipose Tissue Homeostasis in the Context of Beigeing

**DOI:** 10.3390/cells8080831

**Published:** 2019-08-05

**Authors:** Virag Vas, Tamás Háhner, Gyöngyi Kudlik, Dávid Ernszt, Krisztián Kvell, Dániel Kuti, Krisztina J. Kovács, József Tóvári, Mária Trexler, Balázs L. Merő, Bálint Szeder, Kitti Koprivanacz, László Buday

**Affiliations:** 1 Institute of Enzymology, Research Centre for Natural Sciences, Hungarian Academy of Sciences, 1117 Budapest, Hungary; 2 Institute of Physiology, Medical School University of Pécs, 7622 Pécs, Hungary; 3 Department of Pharmaceutical Biotechnology, Faculty of Pharmacy and Szentagothai Research Center University of Pécs, 7622 Pécs, Hungary; 4 Laboratory of Molecular Neuroendocrinology, Institute of Experimental Medicine, 1083 Budapest, Hungary; 5 Department of Experimental Pharmacology, National Institute of Oncology, 1122 Budapest, Hungary; 6 Department of Medical Chemistry, Semmelweis University Medical School, 1094 Budapest, Hungary

**Keywords:** WAT browning, beige adipocytes, adipogenesis, Tks4 scaffold protein

## Abstract

Obesity and adipocyte malfunction are related to and arise as consequences of disturbances in signaling pathways. Tyrosine kinase substrate with four Src homology 3 domains (Tks4) is a scaffold protein that establishes a platform for signaling cascade molecules during podosome formation and epidermal growth factor receptor (EGFR) signaling. Several lines of evidence have also suggested that Tks4 has a role in adipocyte biology; however, its roles in the various types of adipocytes at the cellular level and in transcriptional regulation have not been studied. Therefore, we hypothesized that Tks4 functions as an organizing molecule in signaling networks that regulate adipocyte homeostasis. Our aims were to study the white and brown adipose depots of Tks4 knockout (KO) mice using immunohistology and western blotting and to analyze gene expression changes regulated by the white, brown, and beige adipocyte-related transcription factors via a PCR array. Based on morphological differences in the Tks4-KO adipocytes and increased uncoupling protein 1 (UCP1) expression in the white adipose tissue (WAT) of Tks4-KO mice, we concluded that the beigeing process was more robust in the WAT of Tks4-KO mice compared to the wild-type animals. Furthermore, in the Tks4-KO WAT, the expression profile of peroxisome proliferator-activated receptor gamma (PPARγ)-regulated adipogenesis-related genes was shifted in favor of the appearance of beige-like cells. These results suggest that Tks4 and its downstream signaling partners are novel regulators of adipocyte functions and PPARγ-directed white to beige adipose tissue conversion.

## 1. Introduction

Obesity and obesity-related diseases are becoming increasingly common, and while the obesity-inducing effects of physical inactivity and poor eating habits are well known, other factors that contribute to the development of obesity and obesity-associated conditions are less clear [1]. Therefore, the molecular and genetic mechanisms governing obesity are intensively studied, with the goal of improving the management and treatment of obesity-associated diseases [2,3,4]. Such studies have identified numerous biochemical factors that control fat storage [5,6,7] and differences in fat depot physiologies [8], with the ultimate goal of developing novel strategies to regulate adipose tissue homeostasis.

Tks4 (tyrosine kinase substrate with 4 SH3 domains), which is encoded by the *Sh3pxd2b* gene, belongs to the “positive regulation of adipose tissue development” gene ontology group (GO:1904179) [9]. Tks4 is a scaffold protein that modulates several cellular signaling pathways by bringing regulatory proteins, kinases, and actin-organizing structures into close proximity [10]. Tks4 is involved in podosome formation, cell migration, mesenchymal stem cell differentiation, and bone trabecular formation [11,12,13,14]. Homozygous mutation of *Sh3pxd2b* can lead to the rare genetic disease Frank-ter Haar syndrome (FTHS, OMIM:249420) [15], which affects several tissues due to the defect of Tks4. This disease is characterized by bone abnormalities, such as kyphosis and craniofacial deformities, cardiac disease due to valve or septal defects, glaucoma, and in some cases low adipose tissue depot weight [15,16,17]. Loss of Tks4 has recently also been shown to disturb bone formation, ultimately leading to osteoporosis [18].

Tks4 has also been linked to adipocyte biology, as the protein was initially named FAD49 (factor for adipocyte differentiation 49) and was proposed to be a novel factor that played “a crucial role in the immediate early stage of adipocyte differentiation” [19]. Experiments with the 3T3-L1 preadipocyte cell line showed that the FAD49 mRNA expression level increases during the induction of adipogenesis and that preadipocytes with silenced FAD49 expression do not properly accumulate lipid droplets. In addition to in vitro experiments, animal studies have also demonstrated that Tks4 expression is altered in obesity-related model systems. For example, compared to its status in healthy animals, the Tks4-encoding gene is hypomethylated and transcriptionally upregulated in the inguinal white adipose tissue of diet-induced obese mice [9]. Another analytical study that measured the transcriptome/methylome profile of a fatty pig strain during the fat deposition period identified the Tks4-encoding gene as a “candidate intermuscular fat deposition gene” [20]. Some findings have suggested that Tks4 is also potentially important in human adipocyte biology. Arner et al. demonstrated that the methylation and expression patterns of *Sh3pxd2b* are altered in the white adipose tissue (WAT) of insulin-resistant and insulin-sensitive women [21]. Furthermore, Tks4 mRNA is expressed in human adipose-derived stem cells isolated from the stromal vascular fraction (SVF) of abdominal lipoaspirates [22]. A role for Tks4 in adipocyte differentiation was also suggested by one of our previous studies, in which we showed that mesenchymal stem cells isolated from the bone marrow of Tks4 mutant mice had reduced in vitro adipogenic differentiation potential [14]. Although we have some knowledge of the involvement of Tks4 in adipocyte biology, the cellular roles of Tks4 in different types of adipose tissues and its effects on transcriptional regulation have not been described.

Adipose tissue consists of at least three types of metabolically distinct adipocytes: white, beige, and brown [23,24]. While the classical white fat adipocytes are specialized in synthesizing triglycerides and storing them in the form of lipid droplets, brown adipocytes are responsible for heat production and thermoregulation via their ability to uncouple respiration on the mitochondrial membrane and to dissipate chemical energy via UCP1. The third type of adipocyte, brown-like fat cells (also referred to as beige cells), have been found in WAT [25]. During WAT “browning” or “beigeing”, the UCP1 expression level is elevated, and adipocytes containing several lipid droplets appear among the unilocular white fat cells [26]. This phenomenon can be observed after cold exposure or upon adrenergic stimulus. Recently, several genes have been identified as genetic regulators of beigeing [27,28], and their effects on WAT beigeing have been confirmed in mouse models [25,29]. In addition to various types of adipocyte cells, adipose depots (ADs) also consist of preadipocytes, adipose tissue stem cells, pericytes, endothelial cells, fibroblasts, and macrophages [30]. The cellular compositions of ADs located in different anatomical sites vary, and the specific composition determines the main function of a given AD [8,31].

To identify the roles of Tks4 in adipocyte homeostasis, we analyzed the WAT depots and brown adipose tissue (BAT) of Tks4 knockout (Tks4-KO) mice. We found that in Tks4-deficient WAT cells the adipocyte size was significantly reduced and the number of beige fat cells was increased compared to these features in wild-type (WT) WAT. Furthermore, by measuring the adipogenesis-regulated gene expression signature in WAT from Tks4-KO animals, we found that a loss of Tks4 led to altered expression levels of PPARγ transcriptional targets. 

Therefore, we propose that the Tks4 scaffold protein plays a role in white adipocyte beigeing and that it is a novel regulator of beige cell differentiation.

## 2. Materials and Methods

### 2.1. Animal Studies

The animal procedures in this study were conducted under the approval of the Institutional Animal Ethics Committee (approval number: PEI/001/2042-6/2014), and the currently valid institutional and national guidelines for the care and use of animals were followed. Mice were maintained and handled in accordance with the Guidelines for Accommodation and Care of Animals (European Convention for the Protection of Vertebrate Animals Used for Experimental and Other Scientific Purposes). The Tks4-KO mice (in the C57Bl/6 genetic background) were originally developed by TaconicArtemis GmbH (Cologne, Germany) [14,18]. The fifth and sixth coding exons of the *Sh3pxd2b* gene were flanked by loxP sites, and the floxed exons were then excised via Cre-mediated recombination in the germ line, resulting in inactivation of the *Sh3pxd2b* gene. Tks4-KO mice are infertile: thus, for experiments, heterozygous animals were crossed in every generation to produce Tks4-deficient animals along with their WT littermates. The genotype of each littermate was determined using a previously described genomic PCR method [14]. Western blotting was used to test for the presence of Tks4 protein in various tissue types, including skeletal muscle, heart muscle, brain, lung, spleen, and adipose tissue, and the complete removal of the fifth and sixth exons of *Sh3pxd2b* was confirmed previously in the Tks4-KO mice [14]. As we demonstrated in our previous study, the Tks4 protein is undetectable in the tissues of our Tks4-KO mice. Age-, strain-, and sex-matched genotypically WT and Tks4^−/−^ mice were used in this study.

### 2.2. EchoMRI Measurement

WT and Tks4-KO mice were weighed at the ages of 3–4 months and 7 months. In vivo analyses of their lean body mass, fat mass, and water content were performed in conscious, restrained mice via nuclear magnetic resonance imaging (MRI) (EchoMRI, whole body composition analyzer; E26-233RM, Echo Medical Systems, Houston, TX, USA).

### 2.3. Western Blotting

WT and Tks4-KO mice were euthanized, and interscapular BAT (iBAT), gonadal WAT (gWAT), and subcutaneous WAT (sWAT) samples were removed and stored at −80 °C until further processing. The frozen samples were homogenized with a Potter-Elvehjem Tissue Grinder and lysed in ice cold 30 mM Tris buffer (pH 7.5) containing 100 mM NaCl; 1% Triton X-100, 10 mM NaF; 1 mM EGTA; 1 mM Na_3_VO_4_; 2 mM *p*-nitrophenyl-phosphate; 10 mM benzamidine; 1 mM phenylmethylsulphonyl fluoride (PMSF); and 25 μg/mL each of Pepstatin A, trypsin inhibitor, and aprotinin. The adipocyte lysates were clarified via centrifugation at 14,000 rpm for 10 min at 4 °C twice to remove the pellet and the fat layer. Sample buffer (4x, 0.2 M Tris, 0.277 M SDS, 40% (*v*/*v*) glycerol, 0.588 M β-mercaptoethanol, 0.05 M EDTA, and 1.19 mM bromophenol blue in distilled water) was then added to the clear protein suspension, and the samples were boiled for 3 min. Equal amounts of each sample were subjected to SDS-PAGE using 7.5% running gels. The proteins were then transferred to polyvinylidene difluoride (PVDF) membranes and blocked. The membranes were incubated at room temperature for 60 min with the relevant primary antibody. Polyclonal anti-Tks4-specific antibodies were generated previously [32]. The anti-α-tubulin (DM1A) and anti-UCP1 (u6382) antibodies were obtained from Sigma-Aldrich (St. Louis, MO, USA). The anti-adiponectin (C45B10) antibody was obtained from Cell Signaling Technology (Danvers, MA, USA). After several washing steps, the membranes were treated with horseradish peroxidase-conjugated secondary antibodies (GE Healthcare, Little Chalfont, Buckinghamshire, UK) for 30 min and then washed again. The reacting antigens were visualized with enhanced chemiluminescence (ECL) detection reagents from Amersham Life Sciences Limited (Buckinghamshire, UK).

### 2.4. Immunohistochemical Analysis and Quantification

WT and Tks4-KO mice were euthanized, and BAT, gWAT, and sWAT samples were removed and stored in formalin solution at 4 °C. The fat tissue samples were embedded in paraffin and then sectioned at a thickness of 2 μm. Serial sections were cut and then stained with hematoxylin and eosin (H & E) or subjected to immunohistochemistry. The samples were first deparaffinized in xylene and then hydrated in a series of aqueous alcohol solutions. The sections were treated with H_2_O_2_ to block the endogenous peroxidase activity, and antigen retrieval was performed via heat mediation. The slides were then incubated in protein-blocking solution (Sniper Blocking Solution; Biocare Medical, Pacheco, CA 94553, USA) followed by an overnight incubation at 4 °C with rabbit polyclonal anti-UCP1 primary antibody (u6382, Sigma). The sections were then incubated in Mach 2 Rabbit HRP Polymer (Biocare Medical) as the secondary antibody, and colorimetric detection was performed with AEC (3-amino-9-ethylcarbazole). The immunostained sections were counterstained with hematoxylin. Negative control specimens from the experimental mice were treated in the same way but without primary antibodies. In any case, no background staining was observed. The stained sections were digitally scanned with a Panoramic 250 Flash II high-resolution scanner (3DHISTECH Ltd., Budapest, Hungary). The UCP1 expression pattern and lipid droplet size distribution were quantified in four defined-size fields-of-view per slide by annotating them with CaseViewer 2.1 software (3DHISTECH Ltd., Budapest, Hungary). HistoQuant software (3DHISTECH Ltd., Budapest, Hungary) was used for the histological evaluations.

### 2.5. Cell Isolation, Analysis, and Culture

10–14-week-old Tks4^−/−^ and WT mice were euthanized, and sWAT, gWAT, and iBAT fat pads were isolated, minced, and incubated in digestive medium containing 0.1% (*m*/*v*) (for sWAT and gWAT) or 0.2% (*m*/*v*) (for iBAT) type IV collagenase in Dulbecco’s phosphate-buffered saline (all from Sigma-Aldrich) for 1 h at 37 °C on a shaker. The tissue digestion was stopped by adding an equal volume of fetal bovine serum (FBS), and the resulting cell suspensions were centrifuged twice at 300× *g* for 5 min in Hank’s balanced salt solution (HBSS) (all from Invitrogen, Carlsbad, CA, USA) to isolate the stromal vascular fraction (SVF). The pellets containing the SVF were resuspended in cell culture medium consisting of 10% FBS, 5% horse serum (Invitrogen), 50 U/mL penicillin, 50 μg/mL streptomycin (Sigma-Aldrich), and 2 mM l-glutamine (Invitrogen) in Dulbecco’s Modified Eagle Medium (DMEM)/Ham’s F-12 medium (Life Technologies, Carlsbad, CA, USA). SVF cells were seeded into 6-well plates (VWR, Radnor, PA, USA) at a density of 4 × 10^4^ cells/cm^2^, and the cultures were grown to confluency in a humidified incubator at 37 °C with 5% CO_2_. Confluent cultures were digested and seeded in triplicate into 24-well plates (VWR) for adipogenic induction. During adipogenic induction, the cultures were treated for 7 days with adipogenic medium containing 10% FBS, 0.5 mM 3-isobutyl-1-methylxanthine (Sigma-Aldrich), and 0.1 μM dexamethasone (Sigma-Aldrich) in DMEM/Ham’s F-12 supplemented with 50 U/mL penicillin and 50 μg/mL streptomycin. On day 7, the cultures were fixed with 10% formalin for 20 min at 4 °C and then stained with Oil Red O (Sigma Aldrich) and dimethylmethylene blue. Lipid droplet accumulation was visualized via photographs collected using a digital camera (Nikon Coolpix 4500, Tokyo, Japan) connected to an inverted microscope (Olympus CK2). During osteogenic induction, the cultures were treated for 14 days in osteogenic medium containing 10% FBS, 10 mM β-glycerophosphate (Sigma-Aldrich), 50 μg/mL ascorbic acid (Sigma), and 0.01 μM hydrocortisone (Sigma-Aldrich) DMEM (Life Technologies) supplemented with 50 U/mL penicillin and 50 μg/mL streptomycin. On day 14, the cultures were fixed with 10% formalin for 20 min at 4 °C and then stained with Alizarin Red S (Sigma-Aldrich). Calcium deposition was assessed via images collected with a digital camera (Nikon Coolpix 4500) connected to an inverted microscope (Olympus CK2). The SVF isolation and osteogenic and adipogenic differentiation experiments were repeated twice, and SVF samples from three mice in each group were pooled for each time point. Oil Red O stain accumulation was quantified via ImageJ according to the method of Deutsch et al. [33]. The Oil Red O-positive area was measured in 5–14 fields-of-view per SVF sample at 10× magnification.

### 2.6. Adipogenesis-Regulated Gene Expression PCR Array

WT and Tks4-KO mice were euthanized, and gWAT samples were removed and stored at −80 °C before RNA extraction. The RNeasy Microarray Tissue Mini Kit (cat. no. 73304, Qiagen, Hilden, Germany) was used to isolate total RNA according to the manufacturer’s instructions. On-column DNase-treatment was included to eliminate potential genomic DNA contamination. RNA samples were reverse-transcribed to produce cDNA using the RT^2^ First Strand Kit following the manufacturer’s instructions (cat. no. 33041, Qiagen). 

A Mouse Adipogenesis RT^2^ Profiler PCR Array (PAMM-049ZA, Qiagen) was used for precise gene expression profiling following the manufacturer’s instructions. This array simultaneously measures the expression levels of 84 adipogenesis-related genes. The transcripts and their acronyms are provided in Table S1. The adipogenesis array supports the parallel use of five housekeeping genes (*β-actin*, *β2-microglobulin, Gapdh*, *β-glucorunidase*, *Hsp90ab1*) for accurate normalization and to exclude the presence of genomic DNA contamination. The PCR plates were run on an HT7500 platform, and the results were analyzed online using RT^2^ Profiler PCR Array Data Analysis software (SABioscience). The ∆∆CT method was used for relative quantification. To calculate the fold changes (increases and decreases), the gene expression levels measured in the Tks4-KO samples were normalized to the expression levels in WT samples. During the normalization step, the values from the WT samples were set to 1. During differentiation, small changes in the expression levels of genes encoding transcription factors have large effects on the outcome of the tightly regulated fate decision. Thus, genes were considered to be differentially expressed when the fold change in either direction was >1.5 (fold change cut-off, *F* > 1.5). Data were collected from two Tks4-KO and two WT samples, and the results are represented on a logarithmic scale. The differentially expressed genes were grouped according to their gene ontology similarities and clustered into pro-adipogenic, anti-adipogenic, and pro-brown functional groups [34]. We extracted the PPARγ target genes from the differentially expressed genes with the help of the PPARgene database, which lists the experimentally verified and computationally predicted PPARγ target genes [35]. The Adipogenesis Regulation Network database (ARN) was used to visualize the network of interacting factors [36]. 

### 2.7. Statistical Analysis

All of the results are presented as the mean ± SEM. Differences between two experimental groups (WT versus Tks4^−/−^) were compared using unpaired Student’s *t*-tests. Graphpad Prism version 5.0 software was used for the statistical analysis, and the differences were considered statistically significant when *p* < 0.05.

## 3. Results

### 3.1. Absence of the Tks4 Protein Resulted in Altered Adipose Tissue Composition

#### 3.1.1. Phenotypic Description of the Three Adipose Depots in Tks4-KO Mice

**Figure 1:**
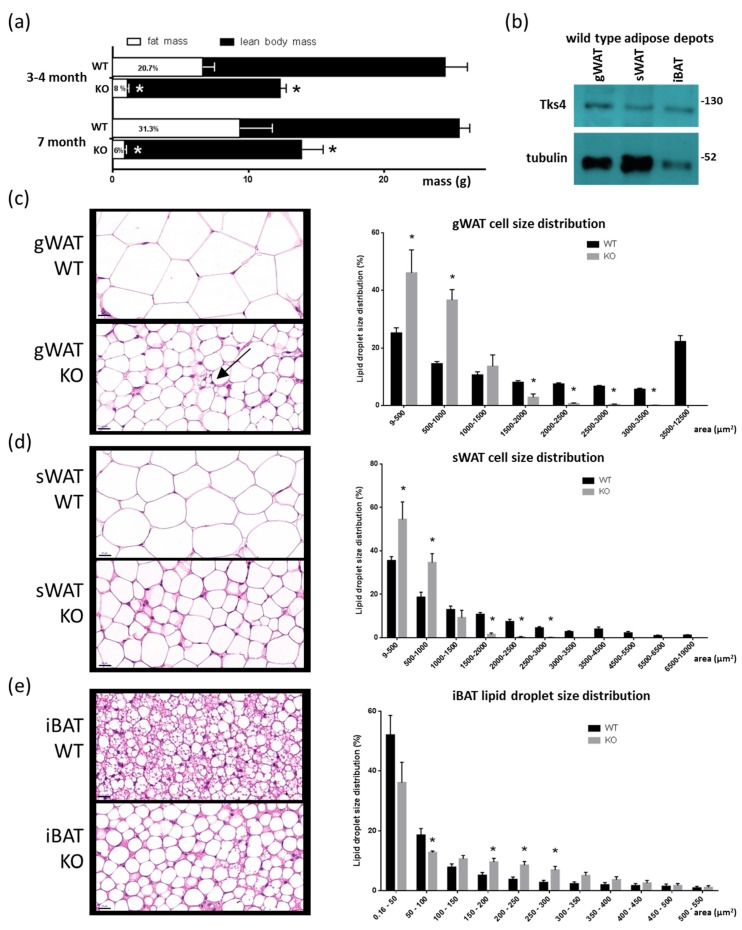
Adipogenic phenotypes of the Tks4-knockout (KO) mice. (**a**) Fat mass and lean body mass of 3–4- and 7-month-old wild-type (WT) and Tks4-KO mice as measured via EchoMRI. The numbers within the white columns indicate the normalized fat mass to body mass percentage. (**b**) Western blot-based detection of Tks4 in WT gonadal white adipose tissue (gWAT), subcutaneous WAT (sWAT), and interscapular brown adipose tissue (iBAT). Tubulin was used as the loading control. Representative hematoxylin and eosin (H & E)-stained (**c**) gWAT, (**d**) sWAT, and (**e**) iBAT sections from WT and Tks4-KO mice are shown on the left side. Scale bar: 20 μm. The arrow indicates a multilocular cell islet among the white adipocytes in the Tks4-KO gWAT. Lipid droplet sizes were quantified, and size distribution diagrams are presented on the right side. Sections of six WT and six Tks4-KO mice were used, and four different fields-of-view from each mouse were analyzed. All data are presented as the mean ± SEM, and *t*-tests were applied to test statistical significance. * indicates *p* < 0.05.

To investigate the roles of Tks4 in adipose tissue, we used Tks4 knockout mice. The Tks4-KO strain was generated in the C57Bl/6 background via targeted disruption of the Tks4-encoding gene *Sh3pxd2b*, resulting in the complete loss of the Tks4 protein [14]. The Tks4-KO mice had reduced fat mass at each tested time point as measured via EchoMRI, indicating a stable lipodystrophy condition (Figure 1A). We further analyzed the levels of Tks4 protein in the different adipose depots in the WT mice and found that Tks4 was expressed in the gWAT, sWAT, and iBAT (Figure 1B). 

We next asked whether the adipocyte morphology was altered in the Tks4-KO mice compared to the WT animals. Hematoxylin–eosin (H & E) staining revealed an obvious decrease in adipocyte size in the gWAT and sWAT of the Tks4-KO mice (Figure 1C,D). Interestingly, the unilocular adipocyte population in the gWAT was accompanied by multilocular cells that resembled beige adipocytes (Figure 1C). In contrast, compared to the BAT of WT animals, the BAT of the Tks4-KO mice exhibited larger lipid droplets and larger adipocytes, and more unilocular white adipocyte-like cells were present in the mutant animals (Figure 1E). As reported previously for WT mice, we also observed that the adipocytes were significantly larger in gWAT than in sWAT [37]. Based on the adipose tissue phenotypes of the Tks4-KO mice, we hypothesized that Tks4 has a yet uncovered role in adipocyte biology.

#### 3.1.2. Tks4-KO and WT Stromal Vascular Fraction (SVF) Cells Shared a Similar De Novo Adipogenic Potential

**Figure 2:**
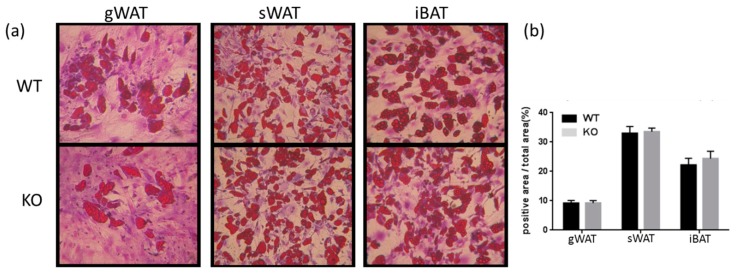
The adipogenic differentiation capacity of stromal vascular fraction (SVF) cells. (**a**) SVF cells isolated from gWAT, sWAT, and iBAT from WT and Tks4-KO mice were treated with adipogenic medium for 7 days and then stained with Oil Red O to visualize the lipid droplets and dimethyl methylene blue to stain the cytoplasm. Representative images (original magnification: 10×) and (**b**) quantification of the Oil Red O staining.

A link is thought to exist between fat cell size and de novo adipogenesis [37,38]. The presence of smaller adipocytes can indicate a population of not fully mature cells that might have originated from a disturbed progenitor/stem cell pool with enhanced differentiation potential [39,40]. Therefore, to assess the differentiation potential of Tks4-KO stem/progenitor cells, WT and Tks4-KO SVF cells were examined to determine whether the decreased adipocyte size in the Tks4-KO mice was due to an altered supply of adipocytes in the WAT. SVF cells were isolated from the three adipose depots, and adipogenic differentiation was induced in the precursor cells in vitro. To assess the presence of stem/progenitor cells in the SVF, we also induced osteogenic differentiation in the SVF cells. The Tks4-KO and WT adipocyte precursors from all three adipose depots showed similar adipogenic and osteogenic differentiation potential (Figure 2B, Figure S1). As previously reported by others for WT SVF, we also observed that the most robust adipocyte accumulation occurred in the sWAT cultures, and SVF cells isolated from gWAT formed the fewest lipid droplet-containing committed adipocytes (Figure 2A). Taken together, these results show that a loss of Tks4 caused a size reduction specifically in white adipocytes accompanied by the appearance of morphologically “beige-like” multilocular cells, without an effect on the overall supply of the adipocyte population.

#### 3.1.3. UCP1 Expression Was Higher in Tks4-KO WAT Than in WT WAT

**Figure 3:**
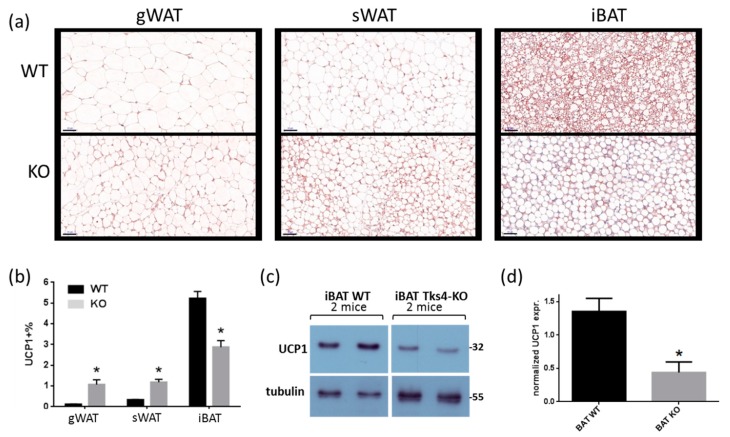
Adipogenic phenotypes of Tks4-KO mice. (**a**) Representative images of uncoupling protein 1 (UCP1)-stained gWAT, sWAT, and iBAT sections isolated from WT and Tks4-KO mice. Scale bar: 50 μm. (**b**) Quantification of the UCP1+ frequency. Sections from six WT and six Tks4-KO mice were analyzed, and four different fields-of-view from each tissue sample were included. All data are presented as the mean ± SEM, and *t*-tests were applied to test statistical significance. * indicates *p* < 0.05. (**c**) Western blot analysis of the UCP1 protein abundance in iBAT isolated from WT and Tks4-KO mice. Tubulin was used as the loading control. A representative blot of two WT and two Tks4-KO iBAT samples is shown. (**d**) Quantification of the UCP1 western blotting results for the iBAT samples isolated from four WT and four Tks4-KO mice from two independent experiments.

The histological observation that Tks4-KO gWAT contained small adipocytes and beige-like cells suggests that the white adipocytes in Tks4-KO mice might undergo browning-like differentiation. Therefore, the expression levels of UCP1, a well-accepted marker associated with brown and beige adipocytes, were measured [41]. Immunohistochemical (IHC) analyses of the different depots revealed that the UCP1 expression level was higher in the Tks4-KO WAT than in the WT WAT (Figure 3A,B). In the WT samples, the UCP1 expression level was lower in the gWAT than in the sWAT, and, as expected, the highest UCP1 expression was detected in the iBAT [42]. Surprisingly, the UCP1 protein level in the Tks4-KO iBAT was lower than that in the wild-type iBAT, as demonstrated via IHC (Figure 3B). The UCP1 protein level in the iBAT samples reached the detection threshold of the western blot assay, which made it possible to confirm that the total UCP1 protein level in the Tks4-KO iBAT was lower than that in the WT iBAT (Figure 3C,D).

### 3.2. Loss of Tks4 Resulted in an Altered Gene Expression Pattern in White Adipocytes

#### 3.2.1. Results of the Adipogenesis-Regulated Gene Expression PCR Array

**Figure 4:**
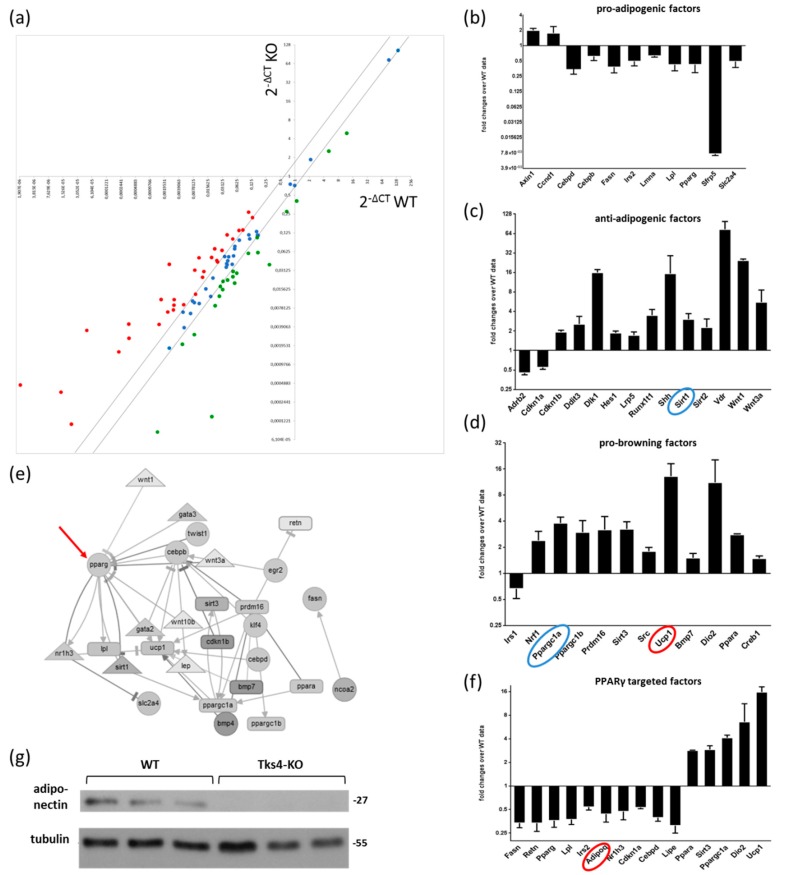
The effects of Tks4 loss on the expression levels of adipogenesis-related regulatory genes. (**a**) Scatter plot showing the up- and downregulation of the investigated gene set. Each dot represents the expression level of a given gene in WT gWAT (the *x* axis represents the WT ΔCT) and in Tks4-KO gWAT (the *y* axis represents the Tks4-KO ΔCT). The two black lines represent the threshold fold change cut-offs at ±1.5. The black lines demarcate the genes with unchanged expression levels (blue dots), and the red-labeled and green-labeled dots represent the up- and downregulated genes, respectively, in the Tks4-KO gWAT (see the gene list in Table S1). The relative expression levels of the genes belonging to the pro-adipogenic factor group (**b**), the anti-adipogenic factor group, (**c**) and the pro-brown factor group (**d**) are shown in separate plots. The blue circles highlight a cofactor (*PPARγC1a*) and a corepressor (*Sirt1*) of PPARγ. The expression levels of the genes marked with red circles were verified via western blotting and immunohistochemical (IHC) analysis. The mRNA levels measured in the WT samples were set to 1, and the fold changes in the gene expression levels in the Tks4-KO samples were calculated. (**e**) An interaction network of the differentially regulated genes. The black arrows represent activating relationships, and the blunt-end lines represent inhibitory relationships among the factors. The red arrow indicates PPARγ as the most highly connected node. (**f**) The relative gene expression levels of the PPARγ-targeted genes. The gene expression fold changes were calculated via the ΔΔCT method. (**g**) The adiponectin protein level was measured via western blotting to validate the change in the mRNA level detected on the array. The adiponectin protein level was measured in three WT and three Tks4-KO gWAT samples, and tubulin was used as the loading control.

To learn more about the role of the Tks4 scaffold protein in the WAT beigeing phenotype, we performed a quantitative mRNA expression analysis of adipogenesis-related genes. As the gonadal depot presented more abundant multilocular beige-like cells and the most pronounced increase in UCP1 abundance among the white adipose depots, we further characterized the gWAT of both Tks4-KO and WT mice. We used the RT^2^ Profiler PCR Adipogenesis Array to measure the expression levels of 84 key genes involved in adipocyte differentiation [43] and five reference genes as internal controls (Table S1, Figure 4A). As suggested by the array manufacturer, we grouped the differentially expressed genes based on their functions during adipogenesis and separately analyzed the expression patterns of pro-adipogenic factors, anti-adipogenic factors and pro-brown differentiation factors. The expression levels of the genes belonging to the pro-adipogenic group were downregulated in the Tks4-KO white adipose depot (Figure 4B). In parallel, the expression levels of the anti-adipogenic factors were elevated in the Tks4-KO mice, suggesting that the classical white adipocyte differentiation process was affected in the absence of Tks4 (Figure 4C). Next, marker genes specific to brown and beige adipocyte differentiation were analyzed. We observed increased expression levels of factors belonging to the pro-browning functional group. Interestingly, the expression levels of *Prdm16* and *PPARγc1a*, the two key beige transcription factors, were also elevated [44,45]. The *Ucp1* mRNA expression level was also increased in the Tks4-KO white adipose depot, as expected based on the IHC data (Figure 3A–D). 

#### 3.2.2. The Differentially Expressed Adipogenesis-Related Genes Formed A Network with PPARγ as Its Central Regulator

Adipogenesis is driven by a series of transcription factor-regulated signaling pathways; thus, we performed a network analysis on the differentially expressed gene set [36]. Out of the list of differentially expressed genes, 28 were present in the Adipogenesis Regulation Network database (ARN) and could be placed in an interaction network diagram (Figure 4E). The network visualization of the relationships between the signaling molecules revealed that the PPARγ adipogenic transcription factor was the most highly connected node. It is well documented that positive and negative regulatory effects converge on PPARγ and that the outcome of the signaling program depends on the balance of these effects [46]. PPARγ, together with its coactivators and corepressors, regulates the expression of several adipogenesis-promoting genes [47] therefore, the expression levels of PPARγ target genes can be used as an indicator to predict the outcomes of the synergistic and antagonistic signals [48]. As shown in Figure 4F, the predicted and validated PPARγ target genes were grouped together, and their expression levels were analyzed [35]. The expression levels of most of the PPARγ target genes were downregulated, including *CEBpd*, *Lpl*, *Lipe*, and *Adipoq*, and notably, these genes function in white adipocytes [49]. In contrast, the highly expressed PPARγ target genes belong to the brown-specific marker gene group [48] (Figure 4F). We concluded that the outcome of the convergent signaling program centered around PPARγ was shifted in favor of the appearance of beige-like cells among the white adipocyte population. 

The gene expression changes detected via the adipogenesis array were validated via western blotting. Nevertheless, it must be considered that the protein levels of the transcription factors are very low and that measuring their levels via western blotting requires a large amount of tissue lysate. Moreover, the isolation of sufficient amounts of protein from the reduced gWAT in the Tks4-KO mice is technically challenging. Therefore, the adipokine adiponectin was chosen as the reference protein for the western blot validation, as it is relatively abundant in normal adipose tissue and is easily detectable via western blotting. Adiponectin was present in the WT gWAT samples but was mostly undetected in the Tks4-KO gWAT, confirming the results of the adipogenic array, which showed reduced *Adipoq* mRNA expression in the Tks4-KO gWAT (Figure 4g).

## 4. Discussion

The formation of beige cells in WAT has been observed in several transgenic mouse strains with a lean, reduced-adiposity phenotype [34,50]. In this study, we collected histological and gene expression data that provided evidence of WAT browning accompanied by a reduced body fat percentage in Tks4-KO mice. 

Silva et al. suggested that adipocyte homeostasis is influenced by the functions of adipose stem cells: therefore, we isolated the stem cell-enriched SVF from Tks4-KO and wild-type mice and compared their capacities for in vitro differentiation [51,52]. We identified phenotypic alterations that are specific to anatomically distinct WAT depots [37,39], as demonstrated by the observation that gWAT-derived SVF cells have a lower adipogenic potential compared to sWAT-derived SVF cells [53,54,55]. We also showed that the adipogenic differentiation potential of the adipocyte precursors in the Tks4-KO SVF was similar to that of the WT cells. These data are inconsistent with the observation that silencing Tks4 mRNA expression in 3T3-L1 cells can block adipogenic differentiation in vitro [19]. These seemingly conflicting results possibly stem from the fact that the SVF consists of a heterogeneous cell population that includes preadipocytes, vascular endothelial cells, pericytes, and adipose tissue mesenchymal stem cells while 3T3-L1 fibroblast cultures represent a homogeneous cell population that originate from a single clone. Therefore, the diverse population of SVF cells might form adipogenesis-supportive microenvironments in vitro and in vivo in conjunction with the extracellular matrix [56], which could rescue the adipogenic differentiation potential of the Tks4-KO preadipocytes. In synchronized 3T3-L1 fibroblast cell lines, the lack of Tks4 and the absence of supportive niche cells resulted in defective adipocyte development [19]. 

Our data also showed prominent gWAT beigeing in Tks4-KO mice relative to the WT mice. Several groups focusing on sWAT have reported a more prominent beige potential in sWAT compared to in gWAT. However, it has also been reported, at least in some cases, that the gWAT depot has a pronounced beigeing phenotype, as has also been published for PDE3B KO mice [57].

Based on the histological examinations and UCP1 expression level measurements, we propose that the WAT of Tks4-KO mice undergoes a partial conversion to beige fat. To further confirm the involvement of the Tks4 protein in the regulation of adipocyte biogenesis, the gene expression profile of a panel of adipogenesis-regulating factors was measured [58]. This analysis revealed that the gene expression pattern was altered in the Tks4-KO mice and that the signaling pathways underlying the beigeing process were more pronounced in these animals than in the WT animals. In the Tks4-KO gWAT, the expression levels of an adipogenesis-supporting gene set tended to be downregulated, while the expression levels of a browning-selective gene set tended to be upregulated. Based on a network visualization generated using the ARN database [36], we found that the differentially expressed genes formed a network and that PPARγ was the central regulator of the included signaling pathways. The PPARγ transcription factor, together with its cofactors, regulates the transcription of several adipogenesis-related genes [45]. PPARγ coactivators, such as the SWI/SNF (mating type switching/sucrose nonfermenting) complex, enhance the transcription of adipogenesis-promoting genes [48]. In contrast, other cofactors (e.g., PPARγC1a) and corepressors (e.g., Sirt1) bind to PPARγ to enhance the expression of several browning-related target genes [59,60,61]. Of note, increased *PPARγC1a* expression was observed in the TKS4-KO gWAT, further supporting the notion that PPARγ-driven gene expression was shifted in favor of the browning differentiation process over the regular white adipocyte differentiation process (Figure 4D). Moreover, the expression level of the *Sirt1* corepressor was also higher in the absence of the Tks4 protein, which could have potentially resulted in the inhibition of PPARγ-mediated transcription of adipogenesis-related genes (Figure 4C). Modification of the transcriptional activity of PPARγ by several cofactors subsequently led to increased or decreased expression levels of its target genes [48]. Therefore, to reveal how the expression levels of PPARγ target genes are regulated in the Tks4-KO mice, we entered the gene list from the adipogenic array into a database of PPARγ target genes [35]. As shown in Figure 4F, the expression levels of browning-promoting PPARγ target genes were upregulated (*Dio2*, *Sirt3*). In contrast, the expression levels of white adipocyte-specific PPARγ target genes were downregulated (*Lpl*, *Lipe*, *Retn*, *Cebpd*). Based on our results, we propose that the loss of Tks4 leads to changes in multiple cellular signaling pathways that contribute to the fine-tuning of PPARγ-regulated transcription in adipocytes.

We also demonstrated that compared to the WT iBAT, the TKS4-KO iBAT had lower UCP1 expression and an increased lipid droplet size, and such alterations could lead to defective BAT function. During this study, we focused on the roles of Tks4 in white and beige adipocyte biology; however, it would also be interesting to study the roles of Tks4 in constitutive brown fat cells. Based on the altered WAT beigeing and the BAT-related phenotypes in TKS4-KO mice, we propose the hypothesis that the formation of beige adipocytes is enhanced in the WAT of Tks4-KO mice as a compensatory mechanism for ineffective BAT cell function. A similar compensatory WAT browning due to impaired BAT function was reported in *Bmpr1a* mutant mice by Schulz at al. [62]. Our hypothesis that an alternative thermogenic beigeing process may be recruited to compensate for altered iBAT function in Tks4-KO mice warrants further examination.

Recently, some data have suggested a positive correlation between BAT function and bone mass [63,64,65,66], indicating that a direct or indirect relationship exists between bone remodeling and brown adipocyte biology. Therefore, it is important to point out that the reduced iBAT size in the Tks4-KO mice [14] was accompanied by an osteoporotic-like bone phenotype [18]. Furthermore, Tks4 has already been identified as a regulator of osteogenic differentiation in BM-MSCs [14]: therefore, the relationship between bone porosity and the altered BAT phenotype identified in this model system is intriguing, and it supports the notion of an interdependence between brown adipocyte function and bone homeostasis.

A better understanding of the mechanisms by which mouse beige adipocytes differentiate in WAT might suggest potential points of intervention relevant in the fight against obesity and obesity-related diseases. It should be noted that mouse models of obesity have limitations, given that several aspects of human obesity differ from those of rodents. Furthermore, different conclusions may arise from mouse and human studies because of differences in the anatomical distribution of fat pads [40], the variant adipocyte composition of BAT [7,8], or the different aging-related changes in the size of the BAT in humans and mice [67,68]. Nevertheless, mouse obesity research is an important and useful tool for gaining insight into mechanisms that govern adipocyte homeostasis that are conserved across species. Therefore, there are growing research efforts to describe brown and beige adipocyte biology and to understand the intersection between mouse and human obesity research [40]. Since it has been shown that cold exposure causes common physiological changes in WAT that lead to adipocyte beigeing in both mice and humans, the use of mouse models could be instrumental in clarifying the mechanism underlying white to beige adipocyte conversion. Based on the results of this study, we propose that Tks4 and its downstream signaling partners are important regulators of adipose tissue homeostasis in the context of beigeing, and our results enhance our understanding of adipocyte browning. However, the translation of Tks4-KO mouse data into targeting Tks4 in human cases for pharmacological obesity therapy requires further studies.

## Supplementary Materials

The following are available online at https://www.mdpi.com/2073-4409/8/8/831/s1, Table S1. Mouse Adipogenesis RT^2^ Profile Array data.
