# Supplementary material for: Analysis of Tks4 Knockout Mice Suggests a Role for Tks4 in Adipose Tissue Homeostasis in the Context of Beigeing
Source: Cells. 2019 Aug 5;8(8):831. doi: 10.3390/cells8080831 (PMC6721678; doi:10.3390/cells8080831)
Supplement: Supplementary file 1 [file cells-08-00831-s001.zip › cells-518607-supplementaryTable 1.pdf]

Table S1

Functional gene-groups:	Symbol	Fold change (RQ)	p value of 2^{-dCT}	Role in PPARγ signaling	Description	Gene name
Pro-adipogenesis related genes:	**Axin1**	2,00	**0,043**		Axin 1	AI316800/Axin/Fu/Kb/Ki/fused/kinky/knobbly
	**Ccnd1**	1,66	0,428		Cyclin D1	AI327039/CycD1/Cyl-1/PRAD1/bcl-1/cD1
	**Lmna**	0,63	**0,009**		Lamin A	Dhe
	**Cebpb**	0,61	0,096		CCAAT/enhancer binding protein (C/EBP), beta	C/EBPbeta/CRP2/IL-6DBP/LAP/LIP/NF-IL6/NF-M/Nfil6
	**Irs2**	0,48	0,185	PPARγ target gene	Insulin receptor substrate 2	Irs-2
	**Slc2a4**	0,48	0,051	PPARγ target gene	Solute carrier family 2 (facilitated glucose transporter), member 4, GLUT4	Glut-4/Glut4
	**Lpl**	0,41	**0,036**	PPARγ target gene	Lipoprotein lipase	-
	**Pparg**	0,41	0,072	PPARγ target gene	Peroxisome proliferator activated receptor gamma	Nr1c3/PPAR-gamma/PPAR-gamma2/PPARgamma/PPARgamma2
	**Fasn**	0,37	0,127	PPARγ target gene	Fatty acid synthase	A630082H08Rik/FAS
	**Cebpd**	0,35	0,059		CCAAT/enhancer binding protein (C/EBP), delta	-
	**Sfrp5**	0,01	**0,019**		Secreted frizzled-related sequence protein 5	AI605071/SARP3
Anti-adipogenesis related genes:	**Vdr**	70,62	0,091		Vitamin D receptor	Nr1i1
	**Wnt1**	24,61	**0,003**		Wingless-related MMTV integration site 1	Int-1/Wnt-1/sw/swaying
	**Dlk1**	15,89	**0,018**		Delta-like 1 homolog (Drosophila)	AW742678/DLK-1/Dlkl/FA1/Ly107/Peg9/SCP1/ZOG/pG2/pref-1
	**Shh**	7,11	0,407		Sonic hedgehog	9530036O11Rik/Dsh/Hhg1/Hx/Hxl3/M100081
	**Wnt3a**	4,72	0,279		Wingless-related MMTV integration site 3A	Wnt-3a/vt
	**Runx1t1**	3,41	0,095		Runt-related transcription factor 1; translocated to, 1 (cyclin D-related)	Cbfa2t1h/ETO/MTG8
	**Sirt1**	2,96	0,099	Coreperessor of PPARγ	Sirtuin 1 (silent mating type information regulation 2, homolog) 1 (S. cerevisiae)	AA673258/SIR2L1/Sir2/Sir2a/Sir2alpha
	**Ddit3**	2,43	0,199		DNA-damage inducible transcript 3	CHOP-10/CHOP10/chop/gadd153
	**Sirt2**	2,13	0,253		Sirtuin 2 (silent mating type information regulation 2, homolog) 2 (S. cerevisiae)	5730427M03Rik/SIR2L2/Sir2l
	**Cdkn1b**	1,93	**0,019**		Cyclin-dependent kinase inhibitor 1B	AA408329/AI843786/Kip1/p27/p27Kip1
	**Hes1**	1,87	**0,021**		Hairy and enhancer of split 1 (Drosophila)	Hry/bHLHb39
	**Lrp5**	1,71	0,113		Low density lipoprotein receptor-related protein 5	BMND1/HBM/LR3/LRP7/OPPG/mKIAA4142
	**Cdkn1a**	0,55	0,290		Cyclin-dependent kinase inhibitor 1A (P21)	CAP20/CDKI/CIP1/Cdkn1/P21/SDI1/Waf1/mda6/p21Cip1/p21WAF
	**Adrb2**	0,45	0,064		Adrenergic receptor, beta 2	Adrb-2/Badm/Gpcr7
	**Adipoq**	0,50	0,207	PPARγ target gene	Adiponectin, C1Q and collagen domain containing	30kDa/APN/Acdc/Acrp30/Ad/GBP28/adipo/apM1
	**Retn**	0,38	0,087	PPARγ target gene	Resistin	ADSF/Fizz3/Rstn/Xcp4
	**Lep**	0,13	**0,038**		Leptin	ob/obese
Adipogen enzyme:	**Lipe**	0,35	**0,037**	PPARγ target gene	Lipase, hormone sensitive	4933403G17Rik/HSL
Pro-Brown Adipose Tissue related genes:	**Ucp1**	12,14	0,158		Uncoupling protein 1 (mitochondrial, proton carrier)	AI385626/Slc25a7/Ucp
	**Dio2**	6,41	0,385		Deiodinase, iodothyronine, type II	5DII/AI324267/DIOII
	**Ppargc1a**	3,76	0,073	Cofactor of PPARγ, PPARγ target gene	Peroxisome proliferative activated receptor, gamma, coactivator 1 alpha (PGC-1a)	A830037N07Rik/ENSMUSG00000079510/Gm11133/Pgc-1α/Pgc1/Pgco1/Ppargc1α
	**Sirt3**	3,19	0,078	PPARγ target gene	Sirtuin 3 (silent mating type information regulation 2, homolog) 3 (S. cerevisiae)	2310003L23Rik/AI848213/Sir2l3
	**Prdm16**	2,93	0,248		PR domain containing 16	5730557K01Rik/csp1/mel1
	**Ppara**	2,82	0,076		Peroxisome proliferator activated receptor alpha	4933429D07Rik/AW742785/Nr1c1/PPAR-alpha/PPARalpha/Ppar
	**Ppargc1b**	2,81	0,314		Peroxisome proliferative activated receptor, gamma, coactivator 1 beta (PGC-1b)	4631412G21Rik/Perc
	**Nrf1**	2,32	0,163		Nuclear respiratory factor 1	C87038/D6Ertd415e
	**Src**	1,80	0,072		Rous sarcoma oncogene	AW259666/pp60c-src
	**Bmp7**	1,50	0,257		Bone morphogenetic protein 7	OP1
	**Irs1**	0,65	0,186		Insulin receptor substrate 1	G972R/IRS-1
Anti-Brown Adipose Tisse related genes:	**Nr0b2**	7,02	0,137	*Cofactor of PPARγ*	Nuclear receptor subfamily 0, group B, member 2	Wnt12
	**Ncoa2**	2,70	0,112	*Cofactor of PPARγ*	Nuclear receptor coactivator 2	Rb/Rb-1/pRb
	**Twist1**	0,58	0,278		Twist homolog 1 (Drosophila)	D1Ertd433e/GRIP-1/Grip1/KAT13C/NCoA-2/SRC-2/TIF2/bHLHe75
	**Nr1h3**	0,54	0,177		Nuclear receptor subfamily 1, group H, member 3	M-Twist/Pde/Ska10/Ska<m10Jus>/Twist/bHLHa38/pdt
	**Wnt10b**	0,44	0,021		Wingless related MMTV integration site 10b	AU018371/LXR/RLD1/Unr1
Pro-White Adipose Tisse related genes:	**Bmp4**	1,97	0,351		Bone morphogenetic protein 4	Bmp-4/Bmp2b/Bmp2b-1/Bmp2b1
	**Klf4**	1,66	0,269		Kruppel-like factor 4 (gut)	EZF/Gklf/Zie
	**Fgf10**	0,45	0,124		Fibroblast growth factor 10	AEY17/BB213776/Fgf-10/Gsfaey17
	**Egr2**	0,36	0,094		Early growth response 2	Egr-2/Krox-20/Krox20/NGF1-B/Zfp-25/Zfp-6
Anti-White Adipose Tissue related genes:	**Gata2**	4,85	0,095		GATA binding protein 2	Gata-2
	**Gata3**	2,41	0,425		GATA binding protein 3	Gata-3/jal

Table S1. Results of the Mouse Adipogenesis RT^2 Profile Array. Fold changes labelled in red indicate upregulated genes while those labelled in green indicate downregulated genes in the Tks4-KO gWAT relative to their levels in the WT animals. The up- and downregulated genes in the table were included in the ARN analysis, the results of which are presented in Figure 3E. The statistical significance of the $2^{-\Delta CT}$ data was assessed via unpaired Student's t-tests.
