# Supplementary figures and images for: Analysis of Tks4 Knockout Mice Suggests a Role for Tks4 in Adipose Tissue Homeostasis in the Context of Beigeing
Source: Cells. 2019 Aug 5;8(8):831. doi: 10.3390/cells8080831 (PMC6721678; doi:10.3390/cells8080831)

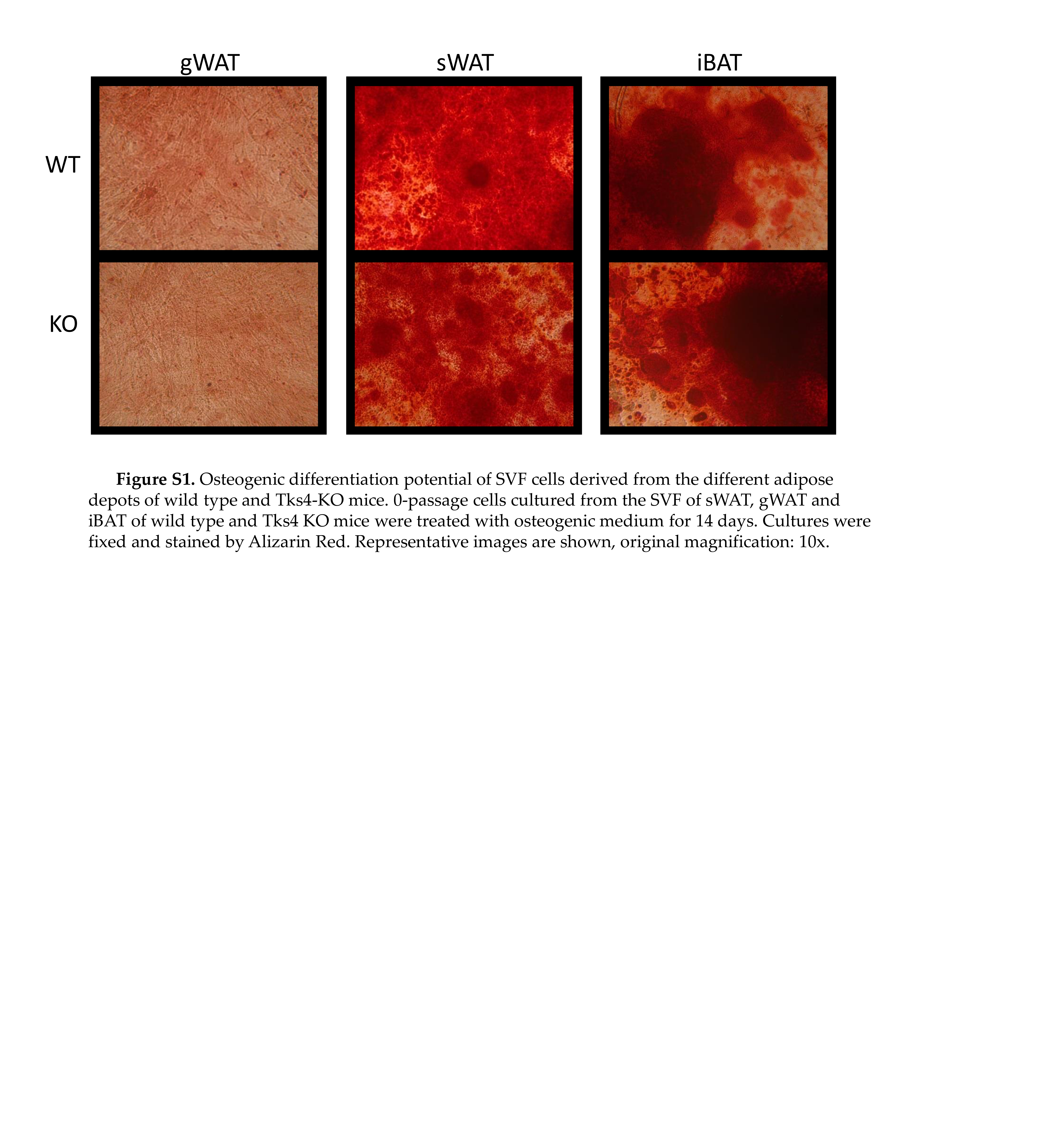

Supplement: Supplementary file 1 [file cells-08-00831-s001.zip › cells-518607-supplementary FigS1.tiff]
